# The Visual Search Strategies Underpinning Effective Observational Analysis in the Coaching of Climbing Movement

**DOI:** 10.3389/fpsyg.2020.01025

**Published:** 2020-05-28

**Authors:** James Mitchell, Frances A. Maratos, Dave Giles, Nicola Taylor, Andrew Butterworth, David Sheffield

**Affiliations:** ^1^Human Sciences Research Centre, College of Life and Natural Sciences, University of Derby, Derby, United Kingdom; ^2^Lattice Training Ltd., Chesterfield, United Kingdom

**Keywords:** eye tracking, think-aloud, sport, education, expertise, gaze behavior, coaching

## Abstract

Despite the importance of effective observational analysis in coaching the technical aspects of climbing performance, limited research informs this aspect of climbing coach education. Thus, the purpose of the present research was to explore the feasibility and the utility of a novel methodology, combining eye tracking technology and cued retrospective think-aloud (RTA), to capture the cognitive–perceptual mechanisms that underpin the visual search behaviors of climbing coaches. An analysis of gaze data revealed that expert climbing coaches demonstrate fewer fixations of greater duration and fixate on distinctly different areas of the visual display than their novice counterparts. Cued RTA further demonstrated differences in the cognitive–perceptual mechanisms underpinning these visual search strategies, with expert coaches being more cognizant of their visual search strategy. To expand, the gaze behavior of expert climbing coaches was underpinned by hierarchical and complex knowledge structures relating to the principles of climbing movement. This enabled the expert coaches to actively focus on the most relevant aspects of a climber’s performance for analysis. The findings demonstrate the utility of combining eye tracking and cued RTA interviewing as a new, efficient methodology of capturing the cognitive–perceptual processes of climbing coaches to inform coaching education/strategies.

## Introduction

Climbing’s acceptance as an Olympic event in Tokyo 2020 is recognition of the sports’ increasing popularity and professionalization (Bautev and Robinson, 2019). As demand increases, so too will the need for effective coaching, thus requiring coach educators to consider how coaching expertise is developed (Sport England, 2018). Climbing coaches employ a range of complex and inter-related strategies to facilitate physical, technical, mental, and tactical improvements (Currell and Jeukendrup, 2008). However, to date, climbing research has predominantly focused on the physiological and the psychological aspects of performance, somewhat neglecting the importance of the technical components of climbing (Taylor et al., 2020). Furthermore, the process by which climbing coaches facilitate technical improvements in their athletes is wholly under-researched.

The characteristics that define expertise in the coaching of climbing movement, and the process by which expertise is developed, have yet to be explored. Wider expertise research has sought to identify the key characteristics of expert performance; among others, one of the key hallmarks that define expert performance is superior visual search behavior (Ericsson, 2017). Research in a variety of sporting contexts (i.e., athletes, officials, and coaches) has demonstrated that experts have a superior ability to pick up on salient postural cues and detect patterns of movement and can more accurately predict the probabilities of likely event occurrences (Williams et al., 2018, p. 663). The superior visual search behavior of expert coaches is thought to be due to more refined domain-specific knowledge and memory structures (Williams and Ward, 2007). Declarative and procedural knowledge, acquired through extensive deliberate practice, enables expert coaches to extract the most salient information from the visual display to identify the key aspects of the athlete’s performance that can subsequently be targeted for improvement (Hughes and Franks, 2004).

Yet without a systematic approach to observational analysis, coaches potentially threaten the validity of their analysis (Knudson, 2013). To understand how coaches analyze and evaluate climbing performance, it is argued that a fundamental step in this process is characterizing the underlying cognitive–perceptual mechanisms that underpin expertise (Spitz et al., 2016). To enable this, the study of expertise in sport has commonly adopted the “Expert Performance Approach” (EPA) (Ericsson and Smith, 1991). In EPA, the superior performance of experts is captured, identifying the mediating mechanisms underlying their performance by recording process-tracing measures such as eye movements and/or verbalizations (Ford et al., 2009). Such advances have begun to enable significant insight into the cognitive–perceptual mechanisms underlying expert performance (Gegenfurtner et al., 2011). For example, lightweight mobile eye tracking devices provide a precise, non-intrusive, millisecond-to-millisecond measurement of where, for how long, and in what sequence coaches focus their visual attention when viewing athlete performance (Duchowski, 2007).

Gegenfurtner et al. (2011) conducted a meta-analysis of 65 eye tracking studies to identify the common characteristics of expert performance. They concluded that the superior performance of experts, across a variety of different domains (sport, medicine, aviation, etc.), could be explained by a combination of three factors: First, experts develop specific long-term working memory skills because of accumulated deliberate practice. Second, expert coaches can optimize the amount of processed information by ignoring task-irrelevant information. This allows for a greater proportion of their attentional resources to be allocated to more task-relevant areas of the visual display (Haider and Frensch, 1999). Finally, they suggest that expert–novice performance differences in visual search are explained by an enhanced ability among experts to utilize their peripheral vision.

To date, however, there has been no eye tracking studies conducted on the visual search strategies of climbing coaches. Yet in other sports, eye tracking technology has yielded insight into differences between expert and novice coaches, which can be used to inform coaching strategies. Here eye tracking research conducted with coaches in basketball (Damas and Ferreira, 2013), tennis (Moreno et al., 2006), gymnastics (Moreno et al., 2002), and football (Iwatsuki et al., 2013) has demonstrated that expert coaches focus on distinctly different locations. Experts fixate their attention on the most salient areas of the visual display as compared to novices (Williams et al., 1999). Additionally, experts demonstrate fewer fixations of greater duration in relatively static tasks/sports (Mann et al., 2007; Gegenfurtner et al., 2011).

Most eye tracking research has, nonetheless, been conducted in laboratory settings, leading some researchers to challenge the ecological validity of the approach (Hüttermann et al., 2018). Adding to this, Mann et al.(2007; see also Gegenfurtner et al., 2011) argue that the more realistic the experimental design is to the realities of the sporting context, the more likely it is that experts will be able to demonstrate their enhanced cognitive–perceptual skills afforded by their increased context-specific knowledge (Travassos et al., 2013). Thus, some researchers have cast doubt on whether the results of laboratory studies can be transferred beyond their immediate context into the complex realities of the coaching environment (Renshaw et al., 2019). Moving forward, therefore, the use of mobile eye tracking technology potentially enables researchers to capture the expert performance of coaches in naturalistic coaching environments, thus enhancing ecological validity and ensuring transferability to coaching practice.

Although eye tracking enables researchers to investigate the processes of visual attention, the relevance of specific gaze location biases to the coaching process still requires elaboration, that is, eye tracking gaze data can tell us *where* someone is looking, but importantly not *why.* Over-reliance on averaged and uncontextualized gaze data potentially oversimplifies and limits our understanding of the coaching process (Dicks et al., 2017). Indeed one of the main conceptual concerns with sports expertise research is the relative neglect of the cognitive processes underpinning expert performance (Moran et al., 2018). As Abernethy (2013) identifies, there remains a lack of evidence on the defining characteristics of sports expertise and how such characteristics are developed. Hence, additional methodological approaches are needed to complement eye tracking if the mechanisms underpinning the superior cognitive–perceptual skills of expert coaches are to be captured.

Currently, two such methodologies are proposed. These are concurrent think-aloud (CTA)—and retrospective think-aloud (RTA). In CTA, the participants verbalize their thought process during the actual task (e.g., Ericsson et al., 1993), whereas in RTA, the participants verbalize their thought process immediately after the task (e.g., Afonso and Mesquita, 2013). In critique, as we can mentally process visual stimuli much faster than we can verbalize our observations, it is argued that, when using CTA, verbalizations are often incomplete (Wilson, 1994). Furthermore, attempting to verbalize complex cognitively demanding tasks while simultaneously performing them affects the user’s task performance and associated gaze behavior (Holmqvist et al., 2011). The alternative, to record participants thinking aloud after the task, circumvents this disruption to the participants’ performance in the primary task. However, due to the time-lag between the primary task and RTA, a “loss of detail from memory or fabulation” may occur (Holmqvist et al., 2011, p. 104). The limitations of RTA are, however, potentially negated when it is combined with eye tracking technology.

Cued RTA utilizes eye tracking gaze data, as an objective reference point to stimulate memory recall, and structure RTA, reducing loss of detail from memory and fabulation (Hyrskykari et al., 2008). Furthermore, cued RTA provides explicit detail as to the declarative and procedural knowledge that underpin the coach’s visual search strategies, adding depth and meaning to otherwise uncontextualized gaze data (Gegenfurtner and Seppänen, 2012). Cued RTA can therefore be adopted for both empirical and theoretical reasons. First, cued RTA is confirmatory in that RTA data enable the researcher to verify the gaze data for accuracy (e.g., fixation location and allocation of attention), and gaze data provide an objective location to reduce memory loss and fabulation when conducting RTA. Second, cued RTA enables the researcher to elicit a greater level of insight into the cognitive–perceptual mechanisms that underpin the visual search strategies of coaches. It is therefore proposed that cued RTA is potentially more effective than either eye tracking or RTA methodologies applied in isolation.

Thus, in the present study, we explored the feasibility and the utility of a novel methodology, combining eye tracking technology with cued RTA, to capture the cognitive–perceptual mechanisms underpinning the visual search behaviors of climbing coaches. As this was a first trial of the combined methodology, three expert and three novice coaches were asked to observe and analyze the live climbing performances of intermediate boulderers in a naturalistic and ecologically valid setting.

## Materials and Methods

### Participants

A total of six UK climbing coaches were recruited for the present study based on their level of expertise (see Moreno et al., 2002). The “expert” group (successful elite, as defined by Swann et al., 2015) consisted of three national team coaches with a minimum of 5 years of professional coaching experience (three males; 8.3 ± 1.5 years). The “novice” group (Nash and Sproule, 2011) consisted of three club-level coaches, with a minimum of 1 year of coaching experience (one female, two males; 3.6 ± 2.1 years). All the participants had normal or corrected-to-normal vision and voluntarily agreed to participate following the local University of Derby ethical approval.

### Materials

#### Climber/Bouldering Problems

The coaches were asked to observe the same intermediate (V4/F6B) climber (male; 21 years) climb four different boulder problems (2 × vertical, 1 × slab, 1 × roof) at a grade of V4/F6B (Draper et al., 2016) at a national center climbing wall. Each boulder problem was repeated three times, requiring the coach to view a total of 12 attempts lasting approximately 16 s each (15.87 ± 0.81 s). The boulder problems were of a maximum height of 4 m and ranged from six to eight moves for each problem. The problems were selected in consultation with an independent national-level coach to ensure that they were judged to be of an appropriate level for the grade and representative of a normal coaching setting.

#### Visual Gaze Behavior

Mobile eye tracking glasses (SMI ETG 2.0; SensoMotoric Instruments, Tetlow, Germany; binocular, 60 Hz) were used to record the coaches’ visual gaze behavior. The gaze data were collected *via* a lightweight smart recorder (Samsung Galaxy 4) using SMI IViewX software. This enabled the recording of visual gaze data in a real-world setting. Prior to capturing eye tracking data, a three-point calibration procedure was implemented by placing three targets in a triangular configuration at a distance of 5 m. The coaches were placed 5 m away from the base of each boulder problem; i.e., at the optimum viewing angle for each specific problem (as decided by an independent national-level coach), and instructed to remain stationary. However, they could move their heads to ensure that the climber remained in the eye-tracker’s recordable visual field. To validate the accuracy, a nine-point calibration grid was placed on each boulder problem, with the markers placed at the outermost areas of the visual field that the coach would be required to observe. This ensured that the gaze data were accurate across the entire visual field. The dependent variable data collected included fixation count, fixation duration, and fixation location.

#### Retrospective Think-Aloud Data Capture

**FIGURE 1:**
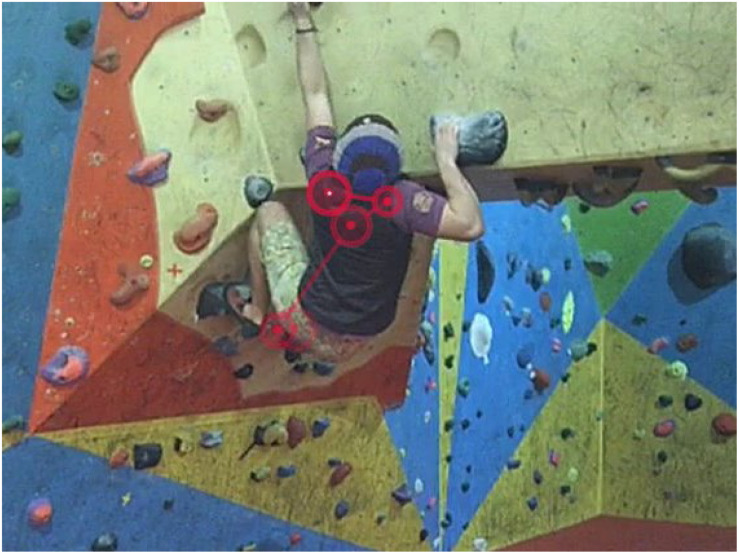
Example of how the gaze data were presented to coaches to cue retrospective think-aloud: visual gaze data super-imposed as 2-s scan path [a connected string of fixations (circles) and saccades (connecting lines)] to cue verbal responses (i.e., why coaches focus on specific fixation locations).

Retrospective think-aloud was conducted using gaze data to cue responses from the coaches: i.e., the coaches were asked to explain individual fixation locations during their analysis of the climber’s performance, verbalizing their relevance to their coaching process. The gaze data were presented to the coach as video replay with the coach’s own visual gaze scan-path super-imposed (see Figure 1). This scan-path showed the most recent 2-s of gaze data appearing to the coaches as a connected string of fixations (circles) and saccades (connecting lines). Each attempt was replayed at 100% speed and then slowed down to 25%.

#### Other Materials

A demographic questionnaire captured the coaches’ prior experience: i.e., highest level of coaching experience, accumulated coaching experience, and current coaching role/responsibilities.

### Procedure

Once the participants had completed the demographic questionnaire, they were fitted with mobile eye tracking glasses and undertook the calibration process. The coaches were then instructed to observe the climber to assess their quality of movement and identify movement errors. It was further explained that they would be required to verbalize their analysis of the climber’s performance later in the experiment. Each coach observed the same climber climb four different boulder problems at a grade of V4, viewing three attempts for each problem. Once each coach had observed all 12 attempts, gaze data were downloaded for further review using SMI BeGaze (V3.2, SensoMotoric Instruments, Tetlow, Germany) analysis software. Using the BeGaze RTA function, the cued RTA interviews were conducted immediately after the collection of gaze data using video replay with the gaze data super-imposed to cue verbal responses. After viewing the gaze data in real time, the participants were asked to scroll through gaze data at 25% speed, explaining why they focused on specific fixation locations and their relevance to the analyses. The fixations discussed were self-selected by the participant in order to reduce researcher bias. The gaze data were replayed until each coach had exhausted all fixations they could recall.

### Analyses

The eye tracking metrics analyzed were: (a) “fixation rate” (i.e., average number of fixations per second), (b) “average fixation duration” (i.e., average fixation duration of all fixations throughout the entire viewing period), and (c) “total fixation duration” (i.e., total duration of a viewer’s fixations landing on a given visual element throughout the entire viewing period) within pre-defined areas of interest. Visual fixations were defined as periods where the eye remained stable in the same location (within 1° degree of tolerance) for a minimum of 120 ms (Catteeuw et al., 2009). The visual gaze data were analyzed using the “semantic gaze mapping” function of SMI BeGaze to manually code fixations against three predefined areas of interest. These were the hands, the feet, and the core regions. Only the gaze data collected while the climber was attempting the problem were included in analysis. As the length of recordings differed for individual coach’s visual gaze behavior due to small variations (±5%) in the athlete’s performance, the data were normalized by cropping the recordings so that each trial was of equal duration to the shortest trial. This enabled the eye tracking metrics (e.g., “total fixation duration”) to be analyzed for comparison between coaches/groups. To enable comparison in visual search strategy, the aggregated gaze data as a function of expert or novice group were used to produce heat maps (Holmqvist et al., 2011). Additional analysis was pursued using Microsoft Excel (Version 15.37, Santa Rosa, CA, United States). Due to the small sample size, the magnitude of differences was determined using Cohen’s *d* (Cohen, 1988).

The cued RTA data were recorded concurrently, ensuring that the interview responses were not separated from the context of the coaches’ individual gaze data. The cued RTA data were transcribed verbatim, and inductive thematic analysis was conducted in accordance to the six-step process outlined by Braun and Clarke (2006). Two members of the research team initially conducted thematic analyses independently before comparing and auditing the analysis process (i.e., first- and second-level codes and final themes). Issues of credibility and transferability were addressed by a process of member checking to ensure a good “fit” between the coaches’ views and the researchers’ final interpretation of themes, as well as ensuring that the themes transfer to the wider coaching context (Tobin and Begley, 2004).

## Results

### Gaze Data

The eye tracking data quality was 98.6% (±0.9), i.e., 98.6% of the samples were captured. An analysis of the gaze data revealed distinct differences between expert and novice groups. The experts demonstrated slower fixation rates (experts 2.23 ± 0.20/s, novices 2.44s ± 0.37/s; *d* = 0.71) and greater average fixation durations (experts 315 ± 30 ms, novices 261 ± 59 ms; *d* = 1.07) than their novice counterparts. In other words, the experts demonstrated fewer fixations but of greater duration.

**FIGURE 2:**
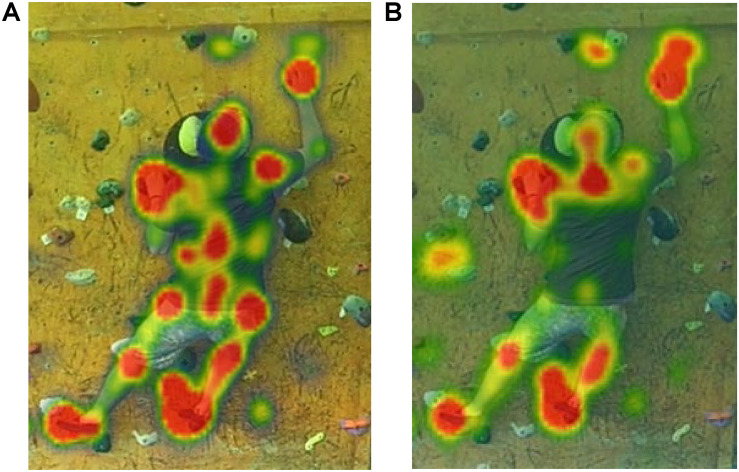
Aggregated heat maps of expert **(A)** and novice **(B)** coaches’ gaze behavior over 12 boulder problems illustrate notable differences in the allocation of visual attention to different regions of the climber’s body.

Furthermore, distinct differences were identified in the locations that the groups allocated attentional resources to. The experts allocated a greater proportion of their attention to the proximal (core) features of the climber’s body, demonstrating a greater number of fixations (experts 58.7 ± 24.5, novices 17.4 ± 1.4; *d* = 2.4) and longer total fixation durations to core body areas (experts 23.6 ± 14.5 s, novices 4.5 ± 1.2 s; *d* = 1.9). The experts additionally placed less attention on the climber’s hand placements than the novices did, with fewer total fixations (experts 41.0 ± 25.9, novices 69.5 ± 27.6; *d* = 1.1) and shorter total fixation durations (experts 16.6 ± 11.6 s, novices 25.8 ± 0.4 s; *d* = 1.1) toward hand placements. Finally, the experts spent more time fixating their attention on the climber’s foot placements than the novices did, with greater numbers of total fixations (experts 44.7 ± 14.6, novices 38.5 ± 14.9; *d* = 0.4) and longer total fixation durations (experts 20.2 ± 4.7 s, novices 11.1 ± 1.4 s; *d* = 2.6) toward foot placements. These differences between the expert and the novice coaches’ visual search strategy were evident from the aggregated heat maps (Figure 2), which illustrate that the experts focused more attention on proximal features (e.g., hips, lumbar region, and center of back), whereas the novices almost solely focused on distal features (e.g., feet and hands).

### Retrospective Think-Aloud Data

The interview durations (min) differed noticeably between the expert and the novice coaches (experts 75.3 ± 12.3, novices 38.0 ± 11.5; *d* = 3.1), reflecting the level of detail that each group was able to provide while explaining their visual gaze data. The thematic analyses revealed three themes: “cognizance of visual search behavior,” “knowledge in the principles of movement and their application,” and “systematic visual search strategy.” Table 1 illustrates the first- and second-level codes that contribute to the three main themes.

**TABLE 1:** Organization of data codes from the thematic analysis.

Themes	Second-level codes	First-level codes
Cognizance of visual search	Ability to relate gaze data to stimulate recall	Recognition of gaze data, enabling distinct recall of visual search strategy
		Unable to recognize own gaze behavior or use gaze location to assist recall of visual search strategy
	Detail in verbalizations	Detailed analysis of rationale for attending to individual/groups of specific fixation locations
		Basic description of rationale for attending to specific fixation locations
Knowledge of principles of movement and their application	Nature/angle of wall in relation to principles of movement	Affordances (i.e., shape, texture, spacing of hold, angles of wall, etc.)
		Types of move/problems
		Rules of thumb for movement
	Naive v’s sophisticated view of movement	Complex relationships
		Isolated/discrete skills
	Limiting factors in climber’s performance	Strength/power in physical performance
		Mobility in performer
		Tactical factors of performance (e.g., route reading)
Systematic visual search strategy	Hierarchy of skill complexity	Techniques and difficulty of order
		“Ticking off” abilities to perform certain actions
		Varying search strategy (i.e., different components of skill)
	Top-down *vs*. bottom-up visual search	Goal-driven visual search
		Stimulus-driven visual search
	Diagnosis of errors	Secondary search to diagnose the cause of symptoms (i.e., of movement errors)
		Knowledge of common errors
		Sequencing and transitioning of movements (i.e., identifying what came before/after)

In respect to the first theme, the expert coaches were far more cognizant of their visual search behavior, being able to verbalize their thought process and provide rationale that explains how the gaze data relate to their coaching process. For example, one expert coach stated:

“I can tell immediately these are my eye movements… You can see I am going through my standard functional movement screening process here. This point here, I am looking at whether hip mobility limiting the climber’s ability to rock-over.” (participant E3)

The novice group, by comparison, was often unable to make any link between their gaze data and their coaching process, simply passing no comment or stating: “I’m not sure why I was looking there” (participant N2). One coach was particularly candid by stating:

“To be honest, I don’t really know what I’m looking for when I’m coaching. I know to look for messy footwork, so that’s what I look for. Beyond that, I don’t know what to look for.” (participant N3)

Considering the second theme, the expert coaches demonstrated a far greater understanding of the principles of movement and their application. Here they demonstrated more complex frameworks and principles of movement that applied to the nature and the angle of the problem. For example, one expert coach succinctly described their process as follows:

“Climbing is a really complex 3D interrelationship between the climber and infinitely varied points of contacts, at differing and changing angles. I try to think of how those points of contact can be used in conjunction, so that the climber can move their center of mass into the optimal position for that particular situation. When the climber is not achieving that position, I try to diagnose secondary factors that may be prohibiting them.” (participant E2)

By comparison, the novice coaches often discussed specific aspects of technique in isolation. For example, participant N1 stated: “So I’m looking for bad footwork here, then I’m looking for if they are holding the hold in the right way.” Comments relating to isolated aspects of technique were common among the novice group with little to no reference to the complex interrelationships between the components of the movement system and their interaction with the environment.

Finally, in reference to the third theme, the expert coaches eluded to a hierarchy of skills that guided their priorities for analysis. Participant E2 observed that:

“If you can see, I am looking at completely different areas during each attempt…looking at different aspects of their performance. I start by looking at the most basic aspects of technique, building up a picture of their ability, working through to more complex skills. When I start to see errors creeping in, I look to see if it is a consistent pattern or just a one-off. If there is a consistent pattern, that is usually the aspect of their climbing I look to address first.”

By contrast, the process of the novice coaches was continually described as a process of search for foot placement errors and search for hand placement errors, continually repeating this cycle. Thus, while both groups eluded to the skills that they prioritized, the above quote highlights how expert verbalizations were more comprehensive and demonstrated a logical/systematic progression in skill complexity. By comparison, novice verbalizations demonstrated a limited and rudimentary grasp of the critical factors that underpin the climbing movement.

## Discussion

Despite the importance of observational analysis in the coaching of climbing movement, the cognitive–perceptual mechanisms underpinning the visual search behavior of climbing coaches have not previously been explored. This study sets out to explore the feasibility and the utility of a previously underutilized methodology within sports expertise research, namely, if mobile eye tracking data, captured in a naturalistic and ecologically valid coaching environment, combined with cued RTA interviews can effectively capture the mechanisms that underpin the visual search behavior in expert and novice coaches. Here the results revealed that the gaze behavior of expert climbing coaches is characterized by fewer fixations, but fixations that were of longer duration than those of novice coaches. Additionally, that experts coaches tend to focus a greater proportion of their attention on proximal regions, whereas the novice coaches typically focused on distal regions. Finally, the RTA analysis revealed that the experts were more cognizant of their visual search strategy, detailing how their visual gaze behavior is guided by a systematic hierarchical process underpinned by complex knowledge structures relating to the principles of climbing movement.

A major finding of the current research was that visual attentional strategies differed between expert and novice climbing coaches. We observed that the expert coaches demonstrated fewer fixations—but these were of greater duration, suggesting that the accumulated context-specific experience of the expert coaches enables them to develop a more efficient visual search behavior. The expert coaches selectively attend to only the most task-relevant areas of the visual display, requiring them to make fewer fixations (of longer duration) to efficiently extract relevant information from specific gaze locations (Ericsson and Kintsch, 1995; Haider and Frensch, 1999). These findings accord with previous studies investigating the visual search strategies of coaches in similar self-paced individual sports (e.g., coaching a tennis serve; Moreno et al., 2006).

The current research further highlighted the relevance of specific fixation locations to more efficient visual search. The proportion of attentional resources that coaches allocated to specific locations varied distinctly between experts and novices. The experts spent nearly five times as long focusing on the proximal regions of the climber’s body (or core) as compared to the novice coaches (refer again to Figure 2), supporting Lamb et al. (2010) notion that the observational strategies of coaches may be overly influenced by the motion of distal segments due to the greater range of motion and velocities than that of proximal segments. It is therefore proposed that the climber’s core represents one of the most salient areas upon which to analyze a climbing performance. Fluency of the center of mass, as defined by the geometric index of entropy, has been shown to be an important performance characteristic (Cordier et al., 1994; Taylor et al., 2020). Identifying the most salient areas to analyze a climbing performance may provide a viable means to inform future coach training, helping novice coaches make their visual search behaviors more efficient (Spitz et al., 2018). However, identifying gaze location alone is of limited practical value to developing coaches unless its relevance is made explicit (Nash et al., 2011).

The addition of cued RTA to the eye tracking methodology revealed three themes that provide insight into the cognitions underpinning the visual attentional strategies of novice *vs*. expert coaches. First, the expert coaches were far more cognizant of their visual search behavior, providing a far more explicit rationale for how their gaze data related to their coaching process. The inability of novice coaches to recall and elaborate on their visual gaze data suggests a randomized and inefficient visual search strategy, that is, they were unclear as to *why* they fixated on specific locations or *what* information they hoped to acquire by doing so. Second, the experts were able to provide rich descriptions of the critical factors that underpin successful movement and relate such principles to their gaze data. Here they demonstrated more complex frameworks and principles of movement applied to the nature and the angle of the problem. Comparatively, the novice coaches provided very little detail on how principles of movement guide their visual search, suggesting that a lack of knowledge regarding the critical factors that underpin climbing movement may be a key factor that limits the effectiveness of their observational analysis. Finally, the experts were more proactive and systematic in their analysis, with their visual search strategy underpinned by a hierarchy of skills (Gegenfurtner et al., 2011). It is likely that the lack of a systematic approach to observational analysis observed among novice coaches potentially limits the validity and the effectiveness of their analysis (Knudson, 2013).

Based on the insights above, it is proposed that the use of cued RTA interviews potentially offers a deeper insight into the cognitive–perceptual process of coaches than the use of eye tracking or think-aloud methodologies employed in isolation. By capturing the declarative and the procedural knowledge that expert coaches utilize to guide their visual search strategy, valuable insight is acquired as to the systematic processes that expert coaches employ to analyze a climbing performance, that is, *where* the most salient areas of the visual display are and *why* they are important to the analysis of a climbing performance. Coach educators may be able to utilize such insights to provide developing coaches with a more explicit rationale to guide their visual search, enhancing the efficiency and the quality of their observational analysis.

## Conclusion

In sum, the present results demonstrate the utility of combining eye tracking technology and cued RTA as a methodology for capturing the cognitive–perceptual processes of climbing coaches. In combining these methods, a range of different cognitions and perceptual behaviors were observed as a consequence of coaching expertise. Combining these technologies potentially offers a valid and a reliable method to capture the processes underpinning the observational analysis of a climbing movement. Indeed the same methodological approach could be applied in a variety of coaching contexts. This stated, a number of limitations and recommendations for future research are highlighted. Despite the ecological validity of the present research, the results must be interpreted tentatively given the small sample size. Furthermore, viewing the live performance of a single athlete presents challenges to study repeatability. Researchers will need to weigh the benefits of ecological validity against replicability. Future research would also benefit from exploring whether the visual search strategies of coaches remain consistent with a greater number of athletes of varying ability, anthropometrics, and style. This will help a comprehensive framework for the observational analysis of a climbing movement to be developed.

## Data Availability Statement

The datasets generated for this study are available on request to the corresponding author.

## Ethics Statement

The studies involving human participants were reviewed and approved by the Human Sciences Research Ethics Committee, University of Derby. The participants provided their written informed consent to participate in this study. Written informed consent was obtained from the individual(s) for the publication of any potentially identifiable images or data included in this manuscript.

## Author Contributions

JM, DG, and NT contributed to the design of the study and in data collection. JM and NT performed data analysis and wrote the first draft of the manuscript. JM, FM, DS, DG, and NT revised the manuscript to produce the final draft, which was subsequently reviewed by all the authors.

## Conflict of Interest

DG was employed by the company Lattice Training Ltd. The remaining authors declare that the research was conducted in the absence of any commercial or financial relationships that could be construed as a potential conflict of interest.
